# Queer Ecology in Loïe Fuller’s Modernist Dance and Magnus Hirschfeld’s *Die Transvestiten*

**DOI:** 10.1215/22011919-9962937

**Published:** 2022-11-01

**Authors:** Ina Linge

**Affiliations:** Department of Languages, Cultures and Visual Studies, University of Exeter, UK

**Keywords:** sexology, modernist dance, queer ecology, transvestitism, Magnus Hirschfeld, Loïe Fuller, Henry Cyril Paget

## Abstract

Dance orients the performer’s body toward both environment and pleasure, yet the intersection of environmental and sexual attunement in dance practice remains an underexplored area of research. This article considers how environmental and sexual readings of dance practice can be brought together by proposing a queer ecological approach to modernist dance. Drawing on research in dance studies, feminist and queer science studies, and sexology studies, the article examines the work of Loïe Fuller, an early pioneer of modernist dance, to show how Fuller’s work engages with themes of both sex and nature and consequently introduces environmentally attuned thinking to early twentieth century sexual knowledge production. By examining the parallels and divergences between Magnus Hirschfeld’s early twentieth-century sexological writing about “transvestitism” and Loïe Fuller’s modernist dance, via the copycat dancer Henry Cyril Paget, I show that both dance and sexology rethought the relationship between sex and nature by grappling, to different extents, with a queer vision of nature, where nature loses its explanatory force and moral authority. This reveals the importance of nature and the nonhuman in the production of modern concepts of sex, gender, and sexuality and the important role that dance can play in illuminating the intersection of sex and nature.

In dance practice, the performer’s body is intimately linked to the space and the environment within which it moves. Environmental dance explores the relationship between bodies on stage and a wider environmental ethics, where movement is attuned to a wider ecosystem.^1^ Here, dance goes beyond understanding nature through aesthetic theory and instead approaches nature through sensuous experience and by “habituating non-anthropocentric practices and values.”^2^ Such sensuous experience is key to dance practice, as dance centers the human body but also pushes this body to the boundaries of physical possibility, exploring the limits of the human. Contemporary dance movements explicitly engage with ways in which dance can connect with the nonhuman world, sometimes with the explicit aim to inspire sustainable action.^3^ Dance and choreography become methods for communicating and sensing the urgency of the climate crisis in the Anthropocene.

Dance and performance cultures also offer particularly rich opportunities for the visualization and embodiment of gender and sexuality. In *Dancing Desires*, Jane Desmond shows how dance histories and practices stand in close relationship to histories of sexuality and should be fruitfully considered together, and that the analysis of dance “as a form of material symbolic bodily practice” can advance queer and feminist theories.^4^ Judith Lynne Hanna argues that dance and sex both “involve the language of the body’s orientation toward pleasure,” making both inseparable.^5^ As a historically specific bodily practice, dance makes visible the relationship between social norms and categorizations and the bodies that inhabit these.^6^ Modernist dance, in particular, puts gender and sexuality center stage.^7^ At the turn of the twentieth century, dance as well as cinema as “art of motion” represented the ambiguous role of women as both spectacle for the male gaze and independent, sexual agents claiming both stage and attention.^8^ Here dance’s engagement with themes of gender and sexuality fits into larger cultural and scientific efforts to understand changing gender roles under the guise of the New Woman. In scientific as well as aesthetic explorations of female sexuality, femininity became a slippery concept, and fears of women as masculinized merged with anxieties about the third sex, a term that can be considered to include a variety of LGBTQ+ identities.^9^

If dance is centrally oriented toward both environment and pleasure, the intersection of environmental and sexual attunement in dance practice remains an underexplored area of research.^10^ In this article, I want to consider how environmental and sexual readings of dance practice can be brought together by proposing a queer ecological approach to modernist dance. Specifically, this article focuses on the work of Loïe Fuller, an early pioneer of modernist dance. Drawing on research in dance studies, feminist and queer science studies, and sexology studies, I argue that Fuller’s dance engages with themes of both sex and nature and consequently introduces environmentally attuned thinking to early twentieth century sexual knowledge production. I investigate how both dance and the sexual sciences, to different extents, grapple with a queer vision of nature, where nature loses its explanatory force and moral authority.

Sex and nature are uncomfortable bedfellows. Approaching the entangled history of sex and nature from a queer theoretical standpoint, which seeks to problematize essentialities of gender and sexual identity, recourse to nature is often considered reductive and re-essentializing.^11^ Stacy Alaimo argues that, as a consequence, “much queer theory has bracketed, expelled, or distanced the volatile categories of nature and the natural, situating queer desire within an entirely social, and very human, habitat.”^12^ Underlying this turn away from nature is a distinction between nature as inflexible and predetermined and culture as meaningful and productive.^13^ Questioning this dichotomy of nature/culture and human/nonhuman, Elizabeth Wilson argues that it should be possible to consider nature as productive in the same way as culture: “there is no intrinsic orthodoxy to biological matter (… it can be as perverse and wayward as any social, textual, cultural, affective, economic, historical, or philosophical arrangement).”^14^ Here, Wilson counters a gesture that excludes the biological as a site of feminist analysis. Wilson’s new materialist feminist approach argues against understanding the biological as inflexible, deterministic, and controlling, and instead argues that a turn to the biological cannot resolve arguments conclusively but requires sustained analysis.

These queer and feminist provocations rethink the relationship between queerness and the natural and contribute to a queer ecological approach to both sex and nature. In their introduction to the landmark collection *Queer Ecologies*, Catriona Mortimer-Sandilands and Bruce Erickson discuss “knots of inquiry” where queer and ecological issues overlap. The first major knot concerns the intersection between histories of sexuality and ecology as “un/naturalizing the queer.”^15^ They summarize that “in the early twentieth century, sexuality became naturalized; an individual’s sexual desires were recoded as expressions of an inherent sexual condition, and that condition was understood in strongly biologized terms.”^16^ The issue raised here is that such sexual-scientific inquiries understood sex and sexuality in reprocentrist terms, attributing a function of either healthy population growth or control in strongly eugenicist terms.^17^ Mortimer-Sandilands and Erickson write that sexual politics is increasingly naturalized during this period, but that the meaning of “naturalization” was continually contested.^18^ If this is the case, then there is at least a possibility that sexual knowledge production destabilized the “evolutionary narrative that pits the perverse, the polluted and the degenerate against the fit, the healthy, and the natural.”^19^

In this article, I offer a reading of Loïe Fuller’s modernist dance that brings out the queer ecological undertones of her work, which, I argue, propose a queer vision of nature that is not oriented toward naturalization as a method of normalizing queer desire. I trace the resonances of her work in sexual knowledge production in the early twentieth century in Germany, in particular to the sexological work of Magnus Hirschfeld. In doing so, I show how early twentieth-century sexual knowledge production can be read through a queer ecological lens. . By bringing modernist dance, the history of sexuality, and environmental humanities together in this way, I also hope to show how methods from environmental humanities, in particular queer ecology, can shed new light on the history of dance as well as the history of sexuality. At the same time, my findings advance the field of environmental humanities by showing how intricately understandings of nature and the natural are linked to concepts of sex, gender, and sexuality and how we must investigate how concepts of sex and nature developed in entangled networks of aesthetic and scientific production.

## Sexual Nature as Metamorphic Continuum in Loïe Fuller’s Modernist Dance

Loïe Fuller was an internationally known Paris-based American dancer who became famous in the early 1890s for her series of dances, including the *Butterfly Dance, Serpentine Dance*, and *Lily Dance*. During her performances, huge pieces of fabric were twisted and turned around Fuller’s body and light projected onto her dress to create the appearance of a butterfly or flower. Upon realizing her dream of dancing in Paris, Fuller rose to huge and absolute fame based on her performance at the Folies Bergère. By 1913, the year Fuller published her autobiography, she was world-famous, having performed in the United States, Jamaica, Mexico, China, Ireland, Romania, France, Germany, and Austria. Her dance became so popular that Fuller often arrived in new cities to find that copycats of her dance had already preceded her.^20^ Fuller was vexed by such copycat acts and unsuccessfully tried to attain copyright over her dance. At the same time, Anthea Kraut argues that Fuller explicitly disavowed any connection to other dance practices that influenced her choreography, including the skirt dance and Indian Nautch dance, making Fuller herself a kind of copycat dancer.^21^ After Fuller’s death in 1928, Fuller’s female partner of thirty years, Gab Sorère, took over her dance troupe.

The value and importance of Fuller’s work and her contribution to the development of modern dance in America and beyond has been widely documented, but the unique, somewhat understated contribution of her work to contemporary understandings of gender, sexuality, and queerness is an area where further scholarship and retrospective analysis are vital. As I argue in this section, Fuller quite specifically used the transformative power of costume to question the nature of gender and sexuality. Fuller started her career in the cross-dressing role of Jack Sheppard in the eponymous play. A contemporary review noted that Fuller “looks like a boy, as few women do in breeches, and she acts like one, which is still less frequently accomplished.”^22^ As Fuller writes in her autobiography, one little girl who saw Fuller’s performance and met her afterward asked, “Well, why does Jack wear girl’s clothes?”^23^ Here, the young audience member appears to misread Fuller’s gender, but this comment also alludes to widely popularized sexological ideas about homosexuality and trans-identifications as forms of sexual inversion. By the time Fuller published her autobiography in 1913, these theories were more widely popularized and her reference to this misreading might have been a veiled attempt to signal her own homosexuality to the initiated reader.

The transformative power of costume to give expression to sexuality featured as a significant trope early on in Fuller’s career. Fuller tells various stories about how she first discovered her *Butterfly Dance*. In her autobiographical retelling of the origin story of her dance, Fuller first wears the skirt as an improvised costume in a performance of *Quack, M.D.*, where she played a woman hypnotized by her doctor. As she flits around the stage, the audience cries “It’s a butterfly! A butterfly!” and later “It’s an orchid!”^24^ A theater manager who witnesses her performance shortly after calls it “the Serpentine Dance.”^25^ Here, Fuller presents her dance practice as being borne out of a moment of convincing transformation, from Fuller the stage girl to butterfly, orchid, and serpent.^26^ Femininity and its disappearance or disavowal is already central to this transformation. Fuller’s dance embodied the transformative power of dress in the realm of gender.

Fuller’s rise to fame in the 1890s coincided with the art nouveau movement, which produced decorative artwork that combined an interest in nature with erotic sensuality. Fuller scholars have shed light on the central importance of Fuller in the art nouveau movement, with its swirling motion and pastoral design depicting flowers and butterflies, which she not only embodied but also significantly inspired.^27^ Beyond these broader statements that understand Fuller’s dance as bringing to life the organic sculptural and decorative forms of art nouveau, however, I want to propose that Fuller’s dance actively intervened in a more specific concern of the art nouveau movement, which sought to depict erotic nature as amoral, predatory, homoerotic, and androgynous via the figure of the fallen or sinful woman. This is in stark contrast to the pastoral and sanitized version of her dance as simply bringing the decorative style of art nouveau to life. In *Art Nouveau and the Erotic*, Ghislaine Wood describes art nouveau as “dependent on nature not only for its energy and forms, but also on the notion of nature as an amoral realm, distinct from culture, where instinct and bestial urge determined behaviour. Nature was an obvious locus for the free rein of erotic fantasies, and Art Nouveau designers used it to signify sex.”^28^ This sexualized and eroticized understanding of nature was often represented by male artists who focused on biblical motifs that aligned women with sin and seduction. See for example Franz von Stuck’s *The Sin*, showing Eve embraced by a serpent, and Aubrey Beardsley’s depictions of Salome as seductress. Here, nature and nonhuman animals are used to depict the eroticism of woman as part of fin-de-siècle fears about the New Woman and a changing image of modernized womanhood. Such readings of Fuller as temptress and seductress abound^29^.

**Figure 1:**
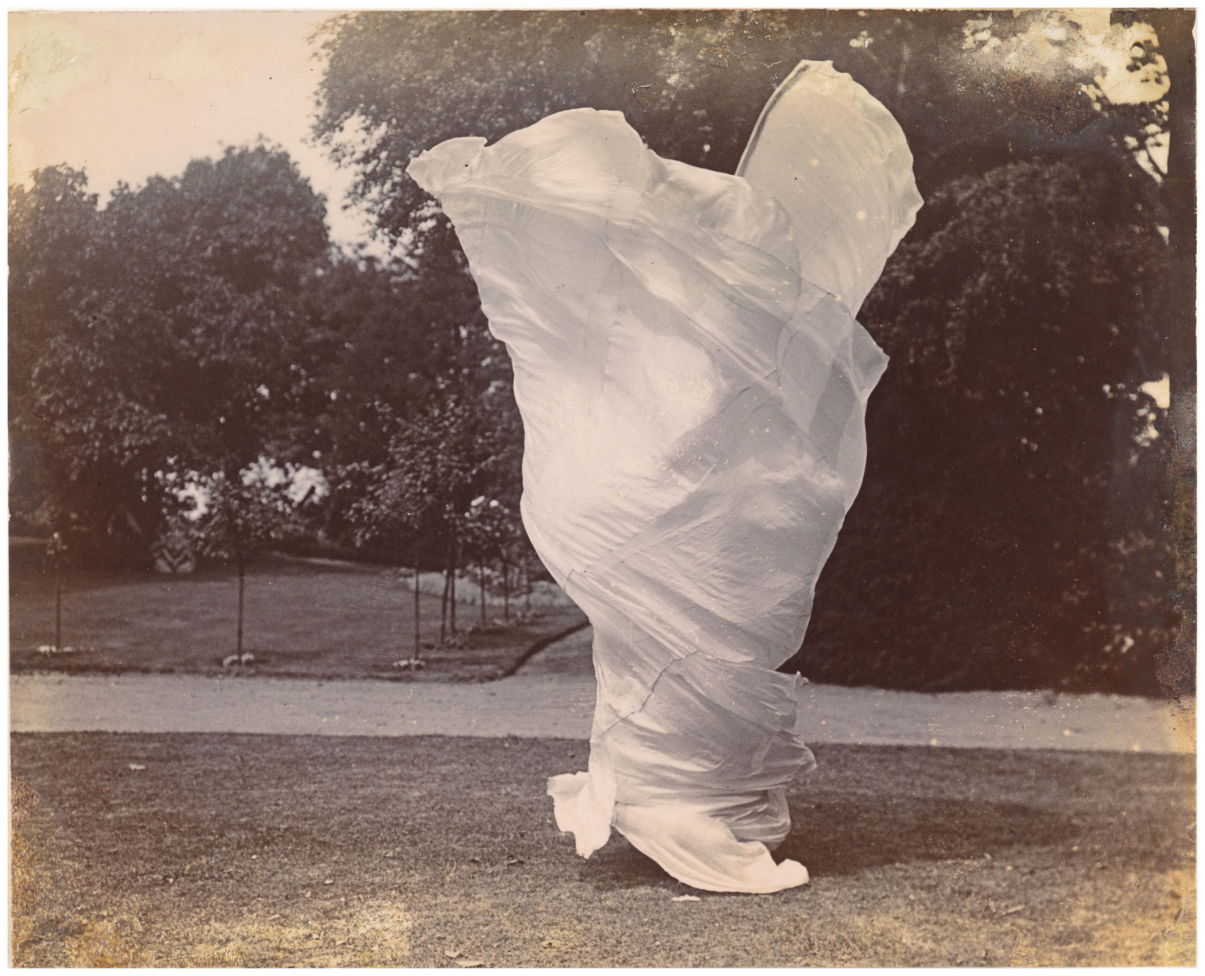
Samuel Joshua Beckett, [Loïe Fuller Dancing] (ca. 1900). Gelatin silver print, 10.1 × 12.5 cm. Gilman Collection, Purchase, Mrs. Walter Annenberg and The Annenberg Foundation Gift, 2005, Metropolitan Museum of Art, New York. Creative Commons license CC0 1.0 (Wikimedia Commons).

Fuller’s dance, I suggest, engages with art nouveau’s concern for women’s erotic nature by reclaiming this amoral and androgynous side of nature as the very place of sexuality. To understand this, we need to first explore how Fuller’s dance expresses sexuality. As contemporary reviewers noted, Fuller’s dances are often based on the complete erasure of the dancer’s body: Fuller manipulated her huge circular dress with long bamboo sticks, which enabled her to lift the material high above her head. At times Fuller’s body disappeared completely (fig. 1). One contemporary reviewer commented that “the vision is never so splendid, so magical, so enrapturing as at the moment when she is about to disappear, to be plunged into nothingness, to be lost in the darkness again.”^30^ This enshrouding of the female body is largely in contrast with the vaudeville performances of the time, which showed women in various stages of undress.^31^ Nonetheless, in both reviews, the body, although almost entirely absent, is the focus of the viewer’s gaze: in one, the focus is on speculations about Fuller’s entire body, now disappearing, whereas in the second example the imminent disappearance becomes a source of rapture. Here, the body offers a moment of final longing and desire precisely because it is about to be withdrawn from view. Fuller’s disappearance teases the viewer to speculate what her “entire body” might look like. As such, it is highly sexualized precisely in its absence. At the same time, the dress becomes the body; as Coffman writes about Stéphane Mallarmé review of Fuller’s performance: “Mallarmé described Fuller’s silky costume as if it were a second skin, playing, as McCarren notes in her translation, off the similarities in sounds between *soi* (herself) and *soie* (silk). The movement of the material was inseparable from the movement of her body.”^32^ Julie Townsend goes even further and sees the “folds and contours of labia” in Fuller’s dress.^33^ One contemporary viewer confirms this vaginal reading of Fuller’s dance: after attending her *Fire Dance*, Belgian symbolist poet Georges Rodenbach writes an enraptured poem in which he uses vaginal imagery to describe Fuller’s dance as a “flower-shaped wound.”^34^

Townsend’s reading of Fuller is particularly important because she links the sexual morphology expressed in her dress to Fuller’s sexuality. Commenting on the materiality of Fuller’s dress and its relationship to the body underneath, she argues that “although her costumes may have given her a means by which to cover and transform herself into an ungendered figure, they may also have been a vehicle for reconfiguring a powerful sexualized identity.”^35^ This powerful sexualized identity is enacted precisely by projecting sexuality into nature. The disappearance of her body, which engenders voyeuristic ways of viewing and sexualizes her dance, also catapults Fuller’s dance into the realm of the nonhuman. In her autobiography, Fuller explicitly comments on the animal nature of her dance, reflecting that in dancing “the human body should, despite conventional limitations, express all the sensations or emotions that it experiences. The human body is ready to express, and it would express if it were at liberty to do so, all sensations just as the body of an animal.”^36^ Fuller’s dance brings the surface of the dress into dialogue with the human body underneath and, in doing so, erodes and confuses this boundary. Images of nature and nonhuman animals, such as butterflies, orchids, and serpents, are central to the execution of such an erosion of boundaries.

Contemporary reviewers understand Fuller as both representing nature and becoming it. When a certain M. Groult shows Fuller his collection of eighteen thousand butterflies, which impressed Fuller greatly, he comments: “These colours *are* you.”^37^ Later on, he adds, with regard to his butterflies: “This is nature as no one can paint it exactly. She has succeeded in it. She is a painter of nature.”^38^ In Gab Sorère’s first review of Fuller’s dance, she writes: “The inanimate becomes animate. … She *is* the butterfly.”^39^ Most famously, Mallarmé reviewed Fuller’s performance in detail, writing:

That is to say the dancer is not a woman who dances, for the combined reasons that she is not a woman, but a metaphor summing up one of the elementary aspects of our form, sword, cup, flower, etc., and that she does not dance, suggesting, through a miracle of shortcuts and dashes, with a bodily writing, what would take in the composition several paragraphs of dialogue or descriptive prose to express: [she is] a poem freed from any instrument of the writer.^40^

In becoming the metaphoric butterfly on stage, Fuller’s dance “abstracts ‘the feminine.’”^41^ Importantly, it does so by abstracting it into nature, at once intimately linked to the body and dress that express such sexual nature but also abstracted in such a way that it can be viewed in its “elementary aspects of form.” At the very metamorphic moment that holds all the sexual energy of the dance—the moment at which the body disappears—the nonhuman also bursts through the human. At the moment where the dancer’s body disappears underneath the silk wings, supported by long bamboo sticks, the dress appears to be animating itself and the dress becomes the body. Between the human and nonhuman, there is no distinction; the human is projected into nature as nature. Importantly, the dance shape-shifts between the two in seamless sequence so that rather than a moment we must speak of a metamorphic continuum. Rather than consisting of a human and nonhuman, or sexualized and desexualized stage of the dance, Fuller’s art of motion queers the binary opposition of both by incorporating them into a continual metamorphosis that absolutely blurs the boundaries between corporeality and incorporeality, human and nonhuman, sexualization and desexualization.

Some viewers inevitably experienced this erosion of boundaries as profoundly unsettling. As Karpenko writes about Fuller’s disappearing figure, it “showcases a highly unstable moment—one in which Fuller appears both human and plant (indeed perhaps more plant than human)—and a moment that could be cast as terrifying.”^42^ This dissolution of boundaries and categories, Coffman argues, was an idiosyncratic modern anxiety.^43^ Between the dancer in her human form, the silk as organic material, and the natural objects evoked, the audience is asked to reflect on the meaning of nature and naturalness with a new sense of sublime wonder and horror.. The natural world is both beautiful and terrifying, knowable and utterly unknowable. Contemporary critics of Fuller’s work described it as “supernatural,” “satanic” and “demonic,” and “witchcraft” and were similarly horrified and captivated by the bestial potential that arose from her work as it transgressed the boundaries between human and nonhuman.^44^

Such readings of Fuller’s dance via dark magic represent both the spectrality of her shape-shifting dance but also incorporate her once more into representations of the New Woman as dangerous seductress via bestial images of nature and the nonhuman. However, by centering her own body on stage in a metamorphic continuum, Fuller’s dance reclaims this amoral and androgynous side of nature as the very site of her own sexuality. In “Acts Against Nature,” Elizabeth Wilson investigates how the concept of nature is used, again and again, to sanitize forms of sexuality that are considered unacceptable. Wilson presents a reading of Laura Aguilar’s photograph (*Grounded #114*) in Dana Luciano’s and Mel Chen’s *Queer Inhumanisms*. The self-portrait shows Aguilar’s body next to a similarly shaped boulder in a desert landscape. Luciano and Chen argue that the inversion of flesh and stone in this photograph also speaks to sexuality: there is something queer in the manner by which conventional boundaries between human and nonhuman dissolve in the image. Based on this dissolution, Luciano and Chen come to an important (but, as Wilson will argue, flawed) political claim about queer sexualities: “To say … that there is no clear division between the natural world and the human body, is also to say that there is no natural law to oppose human deviance.”^45^ This, Wilson argues, erodes the opposition between human and natural world and therefore interrupts the logic of deviance: “there is no foundation or origin … from which certain behaviours and bodies could be said to have strayed. … We can now think of the natural world as an ally for progressive sexual politics.”^46^ But Wilson does not agree with this conflation of nature and neat progressive sexual politics. She argues that Luciano and Chen’s reading “under-read[s] the negativity and confusion that queer entails, and so it renders nature, and the politics we might extract from it, more palatable than perhaps they should be.”^47^ Luciano and Chen project negativity elsewhere in the image—here the desert landscape of the US Southwest, which signifies histories of occupation, displacement, colonization—so that a pastoral vision of an untroubled oneness with the nonhuman, or nature, is maintained.

Wilson reminds us to remain alert to the negativity of nature and the nonhuman, and the negativity of queerness that queer theorists such as Leo Bersani, Lee Edelman, and Jonathan Goldberg have, as it were, unearthed. By focusing on negativity and disorientation, Wilson homes in on the world-shattering potential of sexuality as perversion. In his reading of queer cinema, Cameron Clark proposes a queer antipastoral reading that recognizes “nonegalitarian, inhospitable, and discomforting representations of queerness within the natural world that often struggle to achieve interpersonal or ecological connection.”^48^ This is a queer vision of sexual nature that is significantly different from the “lesbian pastoral” that situates lesbian love as a love for natural landscape and compassion for animals to define it as a privileged and noble identity.^49^ While Wilson’s discussion is less focused on disconnection, Clark’s claim that the queer antipastoral “bring[s] more attention to injurious world-shattering occurrences and their subsequent restructurings”^50^ offers a parallel to Wilson’s queer negative affect in nature. The toxicity of perversion as accusations of indecency, bestiality, ecstatic menace is lost in Luciano and Chen’s vision of a political inhuman, a pastoral vision of sexuality, which reinforces the association between nature and naturalness as mechanisms of acceptability and what Lorraine Daston and Fernando Vidal call the “moral authority of nature.”^51^

In Fuller’s dance we can see this same turning away, or disorientation, in the motion of the *Serpentine Dance* or *Butterfly dance* that reflects a vision of sexuality that is not pastoral, that has not been sanitized. The word *perversity* is etymologically related to *pervertere*, turning away, acts that are deformed, abnormal, awry, and turned the wrong way. This turning motion, this going awry, is also reflected in the word *inversion*, which was commonly used to describe same-sex desire around 1900. If “turning” is the theme of this dance, we are now no longer sure who turns into what, which one comes first, which one is cause, which one effect. This is reminiscent of the Freudian term *displacement*, or *Verschiebung*, where the mind substitutes either a new aim or a new object for goals felt in their original form to be dangerous or unacceptable; or the screen memory, or *Deckerinnerung*, where the most memorable moments from childhood cover up less innocent ones. If displacement and screen memories obstruct the path to the unconscious, then “turning back,” turning the wrong way, leads us back to the unconscious, a place full of negativity and perverse desire.

This is a vision of sexual nature that presents both a turn away from art nouveau’s pastoralism, and from its focus on sinful and fallen women, by presenting a vision of sexual nature that erodes binary oppositions in favor of a metaphoric continuum. Fuller’s vision of sexual nature is deeply disturbing and confounding: the movement of the dress, turning on itself, signifies a return to nature as perversion and inversion; the materiality of the dress highlights the tension between surface and concealment; the boundary between human and nonhuman is unsettled as the face disappears and reappears. Reading Fuller’s work through the lens of queer ecology, as I have done in this section, reveals ways of thinking sex through nature as troubling, disconcerting, unsettling, and haunting. Sexual nature expressed via the metamorphic continuum of Fuller’s dance queers the boundaries between human and nonhuman, the normal and the perverted, nature and culture.

## The Nature of Cross-dressing in Magnus Hirschfeld’s *Die Transvestiten*

Fuller’s work drew the attention of sexologists who were grappling with questions around the naturalness of gender and sexual diversity. In the following section, I explore how the sexologist Magnus Hirschfeld incorporated Fuller’s work into his sexological corpus in order to develop an understanding of natural sexual behavior. Magnus Hirschfeld was a German-Jewish sexologist and founder of the Berlin-based Institute of Sexology (1919–1933). He developed a theory of sexual intermediacy, which claims that Geschlecht (sex/gender) can appear anywhere on a continuous scale between the opposing poles of male and female. Hirschfeld combined his scientific work with lifelong activism against the criminalization of male homosexuality in Germany.

Sexological work often drew on aesthetic production, including literature and visual art.^52^ In addition to this, dance, theater, and costume balls were central to queer culture in Berlin, not just during the heyday of queer Weimar Germany but decades earlier, as Hirschfeld described his forays into the subculture of Berlin’s “third sex” just after 1900, featuring drag performances and private dances.^53^ One depiction of such dance events even forms a pivotal scene in *Anders als die Andern* (*Different from the Others*, 1919), often called the first gay rights film in Germany.^54^

Dance and performance culture offered particularly rich opportunities for the visualization and embodiment of ideas around gender and sexuality.^55^ Dance also formed part of the global investigations of sexology.^56^ Hirschfeld, for example, was fascinated by the Japanese *onnagata*, female impersonators in Kabuki theater.^57^ This interest in global expressions of sexuality led Hirschfeld to form interdisciplinary connections with anthropological and ethnographic research. This led to genuine intercultural and reciprocal exchange, but also tied Hirschfeld and other sexologists into Germany’s colonizing project in parts of Africa and Asia, the perpetuation of German Orientalism, and other forms of racialization, the latter of which Hirschfeld also experienced as a gay Jewish man.^58^

Fuller herself performed in Berlin in 1892 and 1902, the former performance taking place at the Wintergarten variety theater, a center of performance culture in Berlin.^59^ It is no wonder, then, that Fuller’s dance would attract the attention of Berlin-based sexologists. In 1910, Hirschfeld published *Die Transvestiten. Eine Untersuchung über den erotischen Verkleidungstrieb (Transvestites: An Investigation into the Erotic Drive to Cross-dress*). In this important work, Hirschfeld coined the term *transvestitism*, a term that would become a key component of his sexological oeuvre. In his monograph, Hirschfeld mentions the Welsh aristocrat Henry Cyril Paget, the 5th Marquess of Anglesey. What draws Hirschfeld’s attention is the fact that Paget dances in the style of Loïe Fuller’s *Serpentine* or *Butterfly* dance. Hirschfeld then couches Fuller’s and Paget’s dance in the language of transvestitism. In *Die Transvestiten*, Hirschfeld cites at length from an unnamed English newspaper: “Indeed the now twenty-five-year-old nobleman … had *the appearance of a beautiful woman dressed* *in male attire*. … *It was the favourite activity of the young Marquess to perform on the vaudeville stage as a serpentine dancer, an art form in which he was in no way inferior to the gracefulLoie Fuller* “^60^ Henry Cyril Paget, who Hirschfeld presents as having copied Fuller’s dance, was a British aristocrat whose alleged homosexuality led to his inclusion as a case study in various sexological publications. The above passage from *Die Transvestiten* had already appeared in almost identical form in the third volume of the *Jahrbuch für sexuelle Zwischenstufen* (*Yearbook for Sexual Intermediacy*) in 1901, where the marquess is presented as a famous example of aristocratic homosexuality.^61^ In *Das Geschlechtsleben in England (A History of English Sexual Morals*, 1912), sexologist Iwan Bloch once again uses Paget to exemplify famous cases of homosexuality in England.^62^

**Figure 2:**
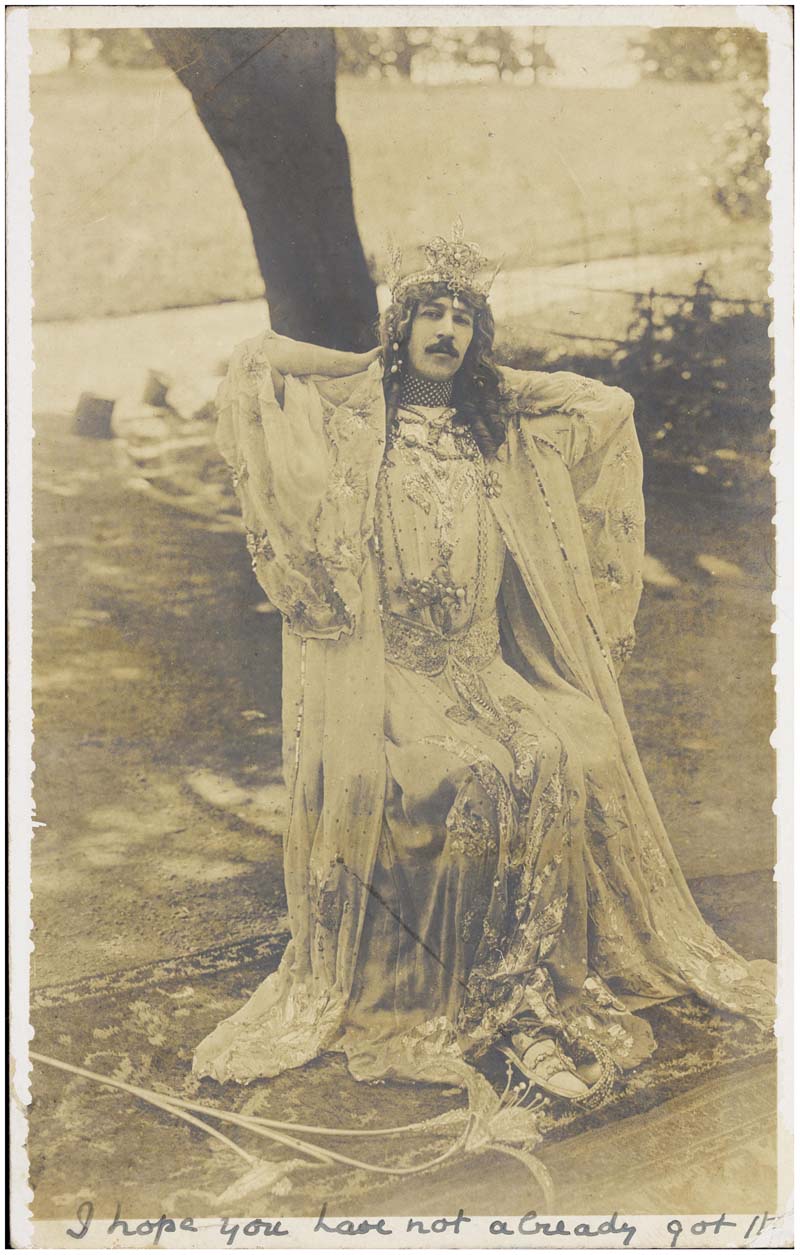
*Henry Cyril Paget, 5th Marquess of Anglesey, seated in drag by a tree*, 1905. Photograph: photoprint; 13.7 x 8.6 cm. No. 2045338i, James Gardiner Collection, Wellcome Library, London, UK (https://wellcomecollection.org/works/hy7v89v2).

Paget was well known for spending a huge amount of his inheritance on costumes and jewelry (see fig. 2). He converted the chapel at his family residence, Anglesey Castle, into a theater called the Gaiety Theatre, where he performed in adaptations of Oscar Wilde’s plays.^63^ Paget’s outrageous and flamboyant lifestyle, his taste for cross-dressing, and the annulment of his marriage due to lack of consummationhave led many to assume that he was gay.^64^ In more overtly homophobic tones, newspapers commented on Paget’s “effemina[cy] to the verge of tears.”^65^ For Hirschfeld, Paget offered a perfect example to show not only that sexual intermediacy was present in working-class individuals but that it affected all people, including royalty and aristocracy.^66^ Connecting sexual intermediacy to aristocracy in this way linked it to respectability, and further supported his aim to make male homosexuality, which was partially criminalized by law, more acceptable.

Although Hirschfeld describes Paget as homosexual in his 1901 *Yearbook*, in 1910 Paget served as a case of “transvestitism.” This definitional shift highlights the importance of Hirschfeld’s *Die Transvestiten*. Late nineteenth-century sexological studies considered both cross-gender identification and same-sex desire under the concept of sexual inversion. Hirschfeld’s work, in particular *Die Transvestiten*, contributes to a larger reconceptualization of gender and sexuality as distinct phenomena.^67^ By the 1920s, the term *transvestite* circulated both within public discourse and within early transgender political organizations.^68^ Those who identified as transvestites made use of such sexological terminology but also pushed against its definitional remit.^69^

Paget’s performance of Fuller’s butterfly dance marks him as a “cross-dresser” in the most explicit way: traditionally, Fuller’s dance was performed by herself and other female copycat acts. The marquess took on a female role by performing the *Butterfly Dance* or *Serpentine Dance*. Viv Gardner suggests that this was a popular theme for Paget—as indeed it was for Fuller. Gardner’s work revealed that the Marquess played with gender reversal in several plays he staged at his Gaiety Theatre, including a performance of *A Runaway Boy* (1901), which featured the marquess as the eponymous runaway boy, a play on *A Runaway Girl* (Seymour Hicks and Harry Nichols, 1898).^70^ His theater itself was likely named after the Gaiety Theatre in London, and Paget’s performance recalls the stage performances of the Gaiety Girls, a dancing corps of young women in fashionable clothing who maintained an air of respectability and politeness as an expression of ideal womanhood and a counterimage to the more sexually revealing burlesque and vaudeville dancers.^71^

This play with such dressing up in different gender roles is key to Hirschfeld’s writing about transvestitism.^72^ In *Die Transvestiten*, Hirschfeld describes the phenomenon as a “Geschlechtsverwandlungstrieb,” literally the drive (*Trieb*) for the transformation or metamorphosis of *Geschlecht* (sex/gender).^73^ Elsewhere in the text, transvestitism is defined as a *Kostümtrieb* (desire to wear a costume)^74^ or, as the title says, *Verkleidungstrieb* (desire to dress up). Hirschfeld’s understanding of transvestitism was in accordance with the then popular theory of the drive as an irresistible urge.^75^ For Hirschfeld, transvestitism designates a specifically erotic desire because, as he argues based on the case material selected, the *Metamorphose allein* (metamorphosis itself) causes libidinal desire.^76^ Borrowing from the language of zoology, metamorphosis relegates transvestitism to the realm of nature, and transvestite metamorphosis parallels the developmental stages of butterflies and moths, in whose sexual development Hirschfeld was highly interested.^77^ Here, Fuller’s dance provides the material for transvestitism: her metamorphosis, as I have shown, heavily relied on the libidinal investment in and the erotic charge of dress and costume.

In his introduction to *Die Transvestiten*, Hirschfeld makes explicit that “transvestitism” is a *natural* part of human sexual variation: throughout, Hirschfeld and his patients refer to their “männliche Natur” (male nature) or “weibliche Natur” (female nature). While “Natur” can be translated as “being,” “essence,” or “character,” I want to argue that it is specifically used to link *Geschlecht* (sex/gender) to nature and naturalness. The development of Hirschfeld’s ideas around sexual intermediacy and transvestitism relied strongly on casting these as natural and therefore immutable phenomena and then using this as evidence that gender and sexuality cannot be changed and will not be affected by criminalization.^78^ In a post-Darwinian context, biologists readily used Darwin’s theory of sexual selection to apply evidence from animal research to human sexual behavior.^79^ Indeed, Darwin’s work and its attention to nonreproductive sexuality has been considered as laying the foundation for modern sexological thought.^80^ Arguments around nature and naturalness remained central to thinking about gender and sexuality during this period.

The meaning of “nature” and “naturalness” in Hirschfeld’s argumentative chainperforms several functions. On an institutional level, grounding sex in nature provided a basis on which to argue for the legitimacy of sexology as science. This included arguing that sexology’s object of study – gender and sexuality – could not be considered immoral, as it was an object of natural-scientific study. According to Hirschfeld and others fighting for homosexual emancipation, this meant that sexuality was fixed, could not be changed by external factors and could therefore not be criminalized. This also ties sexological understanding of nature to a much longer history, reaching back to the pre-modern period, of nature as a form of guidance for morality. As Waltraud Ernst argues, this understanding “conflate[s] the distinction between descriptive statement and moral prescription.”^81^ Looking for guidance from nature then means to conflate “is” with “ought” (Hume) and commit what Lorraine Daston calls “value-trafficking,” projecting values onto nature only to then extrapolate them.^82^ But as Daston points out, “naturalization is in fact a weaker strategy than its critics fear: there are natural orders aplenty to support (or subvert) any and all norms.”^83^ Consequently, the meaning of nature for sexual and gender diversity and its moral authority is far from settled and could be put to different uses.

Hirschfeld, for example, was not alone in focusing on nature as a setting for homosexual emancipation. Max Fassnacht explores how the journal *Die Freundschaft* (*Friendship*) sought to achieve (male) homosexual emancipation by appealing to values of respectability and rights.^84^ In order to achieve this, Fassnacht shows, they appealed to nature and forests, fields, lakes, and mountains as spaces where homosexuality and homoeroticism could be lived free from the constraints of the city. In marshaling nature in this way, they relied on pastoral association between nature, homoeroticism, German nationalism, and Romanticism, thereby proposing “a place in the German national community for a certain type of queer person—masculine men who love each other—at the expense of other sexual, gender, and ethnic minorities.”^85^ In this elite, masculinist approach to homosexuality, nature functions to normalize homosexuality for some, while explicitly excluding theories of the third sex, such as Hirschfeld’s.

Unlike these masculinist and nationalist approaches to nature as particularly German and therefore respectable and aspirational, Hirschfeld’s recourse to nature focused on a much wider spectrum of identities. The central claim of Hirschfeld’s work on sexual intermediate types is that humans are divided into male and female, but that this division is not absolute. On the contrary, many intermediate types between male and female exist. Transvestitism, he argued, is one such type. That such intermediate types are part of natural sexual variation was the crux of Hirschfeld’s sexological theory and the major point of criticism from other sexologists and medical practitioners who understood any deviation from the male and female norm as pathological or abnormal.

Commenting on his observations of sexuality in nature in *Die Transvestiten*, Hirschfeld writes that “as we penetrate more deeply into the innumerable phenomena of nature, nature appears increasingly unpredictable and challenges us to rethink what we know.”^86^ On the one hand, this confirms Hirschfeld’s desire to discover the “laws on nature,” which was also the title of his monograph *Naturgesetze der Liebe*, published the same year that the second volume of *Die Transvestiten* was published, in 1912. Hirschfeld then uses such natural laws to bolster social acceptance and to counter the pathologization of what he called sexual intermediates. Nature here is the focus of a sensationalist discovery of surprising facts about sexuality and desire. Natural phenomena, and here Hirschfeld includes the transvestite as one such phenomenon, can be visually and often anatomically studied in order to yield information about general laws of nature.

On the other hand, this investigation into the natural transvestite also has queerer undertones in that it disrupts existing knowledge and projects an uncategorizable number of sexual expressions onto nature, which, despite Hirschfeld’s efforts, seems to prevent the re-ordering of knowledge. In one example from *Naturgesetze der Liebe*, Hirschfeld discusses the myth of Salome:

No observer can miss the fact that love’s battle can last until the bitter end, not only in the animal kingdom, but also for the cultured person of today. Even if fateful tragedies such as that of Salome are not everyday occurrences, even today some women feel the urge to kiss those dead lips that they were previously denied. This violence of love, this hateful love, stems directly from the ego drive which wants to make the desired object its own.^87^

Here, Hirschfeld attempts to naturalize Salome’s necrophilic desire to kiss the severed head of John the Baptist by showing a similarity in the “Liebeskampf” of humans and nonhuman animals. He understands Salome as sadist but overall tries to show how sadism, too, is part of natural sexual variation. In naturalizing sadistic and necrophilic behavior, however, Hirschfeld instead reveals the unpredictable and challenging results of nature, which he had pointed out two years earlier. Such transgressive forms of necrophilic sexuality that break with human morality run counter to readings of Hirschfeld’s work as privileging order over transgression.^88^ Hirschfeld likely did not intend to create larger social acceptance of necrophiliacs here, but his reference to Salome as sadistic dancer disrupts his mission to order the natural laws of nature.

Another example of how Hirschfeld’s erratic work introduces subtle disturbances is his focus on dress as an expression of nature. Hirschfeld does not claim that dress is the determining factor of transvestitism. Rather, it is one symptom of a larger adoption of a social gender role.^89^ Nonetheless, objects such as dress, hat, veil, jewelry, and so on here become the object of desire and the way by which transvestitism is expressed. This is visualized in the accompanying illustrated volume, published in 1912, where images focus almost exclusively on clothes as costumes.^90^ The volume features “female impersonators” (Damendarsteller, e.g., plate 38), a “male impersonator” (Männerimitatorin, e.g., Vesta Tilley, plate 40) in different social roles and professions (e.g., “women as soldiers,” plate 45), or “male prostitutes” (plate 53). Transvestitism, these images imply, is about the metamorphosis enacted via costume.

Importantly, in Hirschfeld’s understanding, the transvestite links eroticism, dress, and *Geschlecht* to nature. In his conclusion to *Die Transvestiten*, Hirschfeld draws a parallel between human and nonhuman animal behavior that turns on the role of dress. To counteract his readers’ assumptions that military uniforms, with their decorative and colorful details, are anything other than an expression of masculinity, Hirschfeld argues that “in the animal kingdom the male distinguishes itself from the female through the radiance and colour of fur or plumage.”^91^ He then immediately claims that there are examples of the transgression of such gendered coloring—”Haar-oder Federkleid,” literally “hair of feathered dress”—in the animal kingdom. His choice of words creates a parallel between the dress of the transvestite and the fur or plumage of nonhuman animals. Ostensibly, the dress of the transvestite is here naturalized by revealing it to be a natural phenomenon that can be seen across the animal kingdom.

Hirschfeld, however, offers a remarkable example to evidence his claim. He lists Edmond Rostand’s play *Chantecler*, in which the female main character plays a female pheasant who presents herself in the plumage traditionally associated with male pheasants. Hirschfeld adds that such a phenomenon, described by Rostand’s play, appears in not only birds but also insects and most other animal species. Such occurrences, Hirschfeld claims, appear as “transvestite phenomena” but are in fact expressions of “Androgynie,” because they apply only to the physical body, not the psyche (*Seelenleben*).^92^ Throughout *Die Transvestiten*, such a dedication to dress in both body and soul is key to Hirschfeld’s understanding of transvestitism. Earlier in the text, he cautions his readers “that we should not consider clothes as a coincidental, whimsical Thing, as lifeless tissue, but an obvious intentional sign of an inner strive.”^93^ In this referential chain, Hirschfeld at once compares gendered human dress to fur and plumage, then claims that nonhuman animal reversals of such gendered characteristics show the universality—and thus acceptability—of such reversal by using Rostand’s play as evidence, and finally withdraws the comparison by distinguishing *Androgynie* from *Transvestitismus*. Nature’s authority loses its footing in this associative chain. At the same time, Hirschfeld’s conceptualization of transvestitism shows theater and performance to be key components in the creation of sexual knowledge. For Hirschfeld, the performance of the cross-dressing pheasant in *Chantecler* reflects the natural diversity of *Geschlecht* in the animal kingdom.

In drawing on a wide variety of references to aesthetic work in *Die Transvestiten*, from the Salome myth to Rostand’s *Chantecler* and, via Paget, Fuller’s modernist dance, Hirschfeld projects sexual expression onto nature with surprising results. Key to this projection is the sexualized nature of costume, which carries the notion of transvestitism as erotic desire to cross-dress. Via Paget, Fuller’s dress is sexualized precisely by hiding the body underneath, implying a juxtaposition between body and dress but never fully revealing this body. At the same time, the dress becomes a kind of second skin, or fur/plumage, that brings the surface of body and dress into dialogue, eroding and confusing the boundary.

If it is the dress itself that is understood to reveal the transvestite’s innermost sexual nature, such vestral projection defines Fuller’s dance, both literally, as images and light are projected onto her dress, and figuratively, as ideas around femininity and sexuality are projected onto her revolutionary dance by contemporary audiences, critics, and copycat dancers such as Paget. Garelick calls Fuller’s dance “theatre history’s most successful Rorschach test.”^94^ Fuller’s dance could be seen to anticipate sexology’s attempts to project sexual expression onto nature in two ways: Her dance embodies an understanding of sexual nature that turns on its head any binary assumptions about normal and abnormal and instead presents human-animal nature as a metamorphic continuum. But Fuller also anticipates and deflects the voyeuristic, scientific, and penetrative view of sexual nature by counteracting this male gaze on the female body. In a particularly apt counter to depictions of Eve with serpent à la Stuck, Fuller lovingly describes her partner, Gab, as moving like a snake or a young adder.^95^ Here Fuller reclaims misogynistic and homophobic understandings of the sexuality of the New Woman as promiscuous, hypersexualized, and sinful by claiming the snake as embodiment of the New Woman as the very object of female same-sex desire.

## Conclusion

In both Hirschfeld’s sexological work and Fuller’s modernist dance, we can see the emergence of a tentative queer ecological way of thinking. Applying Mortimer-Sandilands and Erickson’s understanding of queer ecology, we can see that both unsettle the separation of “the perverse, the polluted and the degenerate” and “the fit, the healthy, and the natural.”^96^ Sexual knowledge production, via both aesthetic and scientific means, takes place by disrupting “dominant pairings of nature and environment with heteronormativity and homophobia.”^97^ This is enacted not only by inverting this relation—from nature and heteronormativity to nature and sexual diversity—but by dwelling on the complexities and surprises of queer natures and by using nature as a playing field to queer knowledge about gender and sexuality. Hirschfeld uses the natural world to naturalize gender and sexual diversity, but the examples from theater, dance, and performance that he draws on confuse and confound his theoretical framework, as they erode the boundaries between nature and culture, body and soul, human and nonhuman animal. Although this was not the main intention of Hirschfeld’s work, following the traces of Fuller’s dance and other examples of aesthetic production in Hirschfeld’s sexological work shows the disturbing role they played in sexological knowledge production that sought to unearth the nature of sex.

By approaching modernist dance as part of sexual knowledge production via a queer ecological framework, as I have done in this article, I have shown that concepts of nature and the nonhuman were central to the development of modern concepts of sex, gender, and sexuality in turn-of-the-century Germany and beyond. Both aesthetic and scientific inquiries understood nature and the nonhuman as potent yet unpredictable sites to explore emerging ideas about gender and sexuality. To understand the meaning of nature and the nonhuman in this historical context, it is important to consider how these terms have been put to use in scientific and aesthetic production. Through my reading of Hirschfeld and Fuller, the interdisciplinary history of sexuality emerges as a crucial archive for inquiries in the environmental humanities.

## Footnotes

1 Stewart, “Dancing the Face of Place”; Stone, “Environmental Dance.”

2 Stone, “Environmental Dance,” 8.

3 Mosko, “Stepping Sustainably.”

4 Desmond, *Dancing Desires*, 3.

5 Hanna, “Dance and Sexuality,” 212.

6 Desmond, *Dancing Desires*, 4.

7 Ruprecht and Wittrock, “Queering Dance Modernism”; Bland, “Trial by Sexology?”

8 For the role of women in “art of motion,” see Gunning, “Loïe Fuller”; Coffman, “Women in Motion.”

9 On the idea of the New Woman as masculine, see Sutton, *Masculine Woman*.

10 Important work in this area includes Michael J. Morris’s research on dance and ecosexuality and Kelly Perl Klein’s performance-based inquiry into sensuous ecological activism. See Morris, “Matter”; Klein, “Dancing into the Chthulucene”

11 For an excellent and detailed discussion of queer theory’s problem with nature, see Seymour, *Strange Natures*, 3–13.

12 Alaimo, “Eluding Capture,” 51.

13 For a discussion of the nature/culture dichotomy, see Kirby, *Quantum Anthropologies*.

14 Wilson, *Gut Feminism*, 27.

15 Mortimer-Sandilands and Erickson, “Introduction,” 6

16 Mortimer-Sandilands and Erickson, “Introduction,” 8.

17 Mortimer-Sandilands and Erickson, “Introduction,” 9.

18 Mortimer-Sandilands and Erickson, “Introduction,” 6–8.

19 Mortimer-Sandilands and Erickson, “Introduction,” 3.

20 Fuller, *Fifteen Years*, 54.

21 Kraut, “White Womanhood.”

22 The *Times*, cited in Current and Current, *Loie Fuller*, 19.

23 Fuller, *Fifteen Years*, 149.

24 Fuller, *Fifteen Years*, 31.

25 Fuller, *Fifteen Years*, 38.

26 Kraut shows that Fuller explicitly disavows any connection to other dance practices that influenced Fuller’s choreography, including the skirt dance and Indian Nautch dance, to support her claim that she owns the copyright to her *Serpentine Dance*. Kraut, “White Womanhood.”

27 Coffman, “Women in Motion,” 75; Karpenko, “‘Inanimate Becomes Animate,’” 572; Current and Current, *Loie Fuller*, 4.

28 Wood, *Art Nouveau*, 60.

29 See Garelick, *Electric Salome*, 163; Coffman, “Women in Motion,” 87.

30 Current and Current, *Loie Fuller*, 57.

31 Coffman, “Women in Motion,” 76.

32 Coffman, “Women in Motion,” 92.

33 For further queer readings of Fuller, see Latimer, “Loie Fuller”; Townsend, “Alchemic Visions”; Garelick, *Electric Salome*.

34 Garelick, *Electric Salome*, 163.

35 Townsend, “Alchemic Visions,” 91.

36 Fuller, *Fifteen Years*, 70.

37 Fuller, *Fifteen Years*, 132. Emphasis added.

38 Fuller, *Fifteen Years*, 136.

39 Fuller, *Fifteen Years*, 265.

40 Modified by Jones from the translation by Lloyd and Caws, *Mallarmé in Prose*, 109; cited in Jones, “Une ecriture corporelle,” 240.

41 Coffman, “Women in Motion,” 92.

42 Karpenko, “‘Inanimate Becomes Animate,’” 572.

43 Coffman, “Women in Motion,” 77.

44 Griffith, “Loie Fuller,” 540; Fuller, *Fifteen Years*, 265, quoted in Coffman, “Women in Motion,” 91, cited in Current and Current, *Loie Fuller*, 54.

45 Luciano and Chen, “Introduction,” 185.

46 Wilson, “Acts against Nature,” 19-20.

47 Wilson, “Acts against Nature,” 21.

48 Clark, “Grief,” 212.

49 Mortimer-Sandilands and Erickson, “Introduction,” 24.

50 Clark, “Grief,” 212-13.

51 Daston and Vidal, *Moral Authority*.

52 Schaffner, *Modernism and Perversion;* Tobin, *Peripheral Desire;* Lang and Sutton, “Aesthetics of Sexual Ethics.”

53 Hirschfeld, *Berlins drittes Geschlecht*.

54 Linge, “Sexology.”

55 Desmond, *Dancing Desires;* Ruprecht and Wittrock, “Queering Dance Modernism”; Bland, “Trial by Sexology?”

56 On the global reach of sexology, see Bauer, *Sexology;* Fisher and Funke, “‘Let Us Leave’”; Fuechtner, Haynes, and Jones, *Global History*.

57 Herrn, “Magnus Hirschfeld’s Onnagata.”

58 On sexology and race, see Marhoefer, *Racism and the Making of Gay Rights;* Schrader, “Fears and Fantasies”; Funke, “Navigating the Past”; Tobin, “Sexology”; Bauer, “‘Race.’”

59 Current and Current, *Loie Fuller*, 149.

60 Hirschfeld, *Die Transvestiten*, 453-54. Emphasis original. All translations of this volume are my own, unless otherwise noted.

61 “Zeitungsausschnitte,” 543-44.

62 Bloch, *History of English Sexual Morals*, 420–21.

63 Gardner, “In the Eye,” 65.

64 Gardner, “In the Eye.”

65 *St. James’s Gazette*, March 15, 1905, cited in Gardner, “In the Eye,” 70.

66 The same message appears in the sexual enlightenment film *Anders als die Andern (Different from the Others*), to which Hirschfeld also contributed. See Linge, “Sexology,” 8–10.

67 Sutton, “‘We Too Deserve a Place,’” 336.

68 Sutton, “‘We Too Deserve a Place,’” 335-36.

69 Heaney, “‘I Am Not a Friend.’”

70 Gardner, “In the Eye,” 69.

71 Hyman, *Gaiety Years*.

72 For a detailed discussion of *Die Transvestiten*, see Herrn, *Schnittmuster des Geschlechts*.

73 Hirschfeld, *Die Transvestiten*, 167.

74 Hirschfeld, *Die Transvestiten*, 72.

75 Herrn, “Magnus Hirschfeld’s Onnagata,” 383.

76 Hirschfeld, *Die Transvestiten*, 178.

77 Linge, “Potency of the Butterfly.”

78 See, for example, Hirschfeld’s interest in genetics experiments on “intersex” butterflies, which led him to draw conclusions for human homosexuality. Linge, “Potency of the Butterfly.”

79 Milam, *Looking*.

80 Brooks, “Darwin’s Closet.”

81 Ernst, *Histories*, 4.

82 Daston, *Against Nature*, 4.

83 Daston, *Against Nature*, 68.

84 Fassnacht, “On the Ground.”

85 Fassnacht, “On the Ground,” 440.

86 Hirschfeld, *Die Transvestiten*, 1.

87 Hirschfeld, *Naturgesetze der Liebe*, 210. All translation of this edition are my own.

88 Schneider, “‘Pleasure of the Uniform.’”

89 Hirschfeld, *Die Transvestiten*, 162.

90 Hirschfeld and Tilke, *Der erotische Verkleidungstrieb*.

91 Hirschfeld, *Die Transvestiten*, 550.

92 Hirschfeld, *Die Transvestiten*, 551–52.

93 Hirschfeld, *Die Transvestiten*, 258.

94 Garelick, *Electric Salome*, 222.

95 Fuller, *Fifteen Years*, 251.

96 Mortimer-Sandilands and Erickson, “Introduction,” 3.

97 Mortimer-Sandilands and Erickson, “Introduction,” 22.
